# Characterization of anti-soybean agglutinin (SBA) IgY antibodies: a new strategy for neutralization of the detrimental biological activity of SBA

**DOI:** 10.3389/fvets.2024.1382510

**Published:** 2024-04-12

**Authors:** Nancy Ruiz Díaz, Carlos Cisternas, Mauricio Silva, Adrián Hernández, Pablo Chacana

**Affiliations:** ^1^Programa de Doctorado en Ciencias Agropecuarias, Facultad de Recursos Naturales, Universidad Católica de Temuco, Temuco, Chile; ^2^Escuela de Medicina Veterinaria, Facultad de Recursos Naturales y Medicina Veterinaria, Universidad Santo Tomás, Temuco, Chile; ^3^Escuela de Tecnología Médica, Facultad de Salud, Universidad Santo Tomás, Temuco, Chile; ^4^Departamento de Ciencias Veterinarias y Salud Pública, Facultad de Recursos Naturales, Universidad Católica de Temuco, Temuco, Chile; ^5^Núcleo de Investigación en Producción Agroalimentaria, Facultad de Recursos Naturales, Universidad Católica de Temuco, Temuco, Chile; ^6^Departamento de Ciencias Agropecuarias y Acuícolas, Facultad de Recursos Naturales, Universidad Católica de Temuco, Temuco, Chile; ^7^Instituto de Patobiología, Instituto Nacional de Tecnología Agropecuaria, Buenos Aires, Argentina

**Keywords:** soybean, agglutinin, IgY, haemagglutination, antinutritional factor

## Abstract

Anti-soybean agglutinin (SBA) IgY was produced, and its potential to neutralize the haemagglutinating activity of SBA *in vitro* was tested. Thirty-five-week-old hens [treatment (*n* = 5) and control (*n* = 5)] were immunized with SBA or injected with saline 4 times every 15 days. Eggs were collected after the last immunization, and IgY was extracted using the polyethylene glycol (PEG) method. Serum anti-SBA IgY titres in immunized hens increased after the first immunization and reached a plateau between days 45 and 60. In contrast, specific IgY titres in the control group remained at basal levels throughout the evaluation. Average IgY titres were significantly higher in the treatment group on days 15, 30, 45, and 60. Total IgY content in the egg yolk extract was 38.7 ± 1.6 and 37.7 ± 1.5 mg/ml for the treatment and control groups, respectively. The specific anti-SBA IgY titer detected in the egg yolk extract was significantly higher (*p* < 0.001) for hens in the treatment group compared to the control group, with OD_450nm_ values of 0.98 ± 0.05 and 0.058 ± 0.02, respectively. The specificity of anti-SBA IgY was confirmed by the Western blotting, and the inhibition of SBA-induced haemagglutination *in vitro* was compared with D-galactose, a known molecule that binds to SBA and blocks its binding to erythrocytes. The inhibition of SBA-induced haemagglutination by the anti-SBA IgY reached 512 units of haemagglutination inhibition (UHI), compared to 8 or 256 UHI, respectively, when IgY from control chickens or D-galactose was used. Thus, anti-SBA IgY antibodies were efficiently produced in large quantities and effectively inhibited SBA-induced haemagglutination *in vitro*.

## Introduction

Lectins are natural bioactive proteins and glycoproteins, particularly abundant in legumes (1) and seeds (2), with specific affinity to bind simple or complex sugars (3). The most studied lectins are concanavalin A (convA) and soybean agglutinin (SBA), which are the phytohaemagglutinins present in the faba bean (*Canavalia ensiformes*) and soybean (*Glicine max*) (4). SBA has a molecular weight of 120 kDa (5) and consists of four identical tetramers of 30 kDa each (6). Each SBA tetramer consists of an oligosaccharide chain containing *N*-acetylgalactosamine and mannose in its structure (3). In addition, several polar amino acid residues (Lys, Arg, Asp, Thr, Ser, Asn, and Gln) have been described within its polypeptide chains (7) with two specific binding sites for sugars (8).

Soybean is a major protein ingredient in commercial broiler and layer diets, accounting for ~30% of the diet at each stage of production (9). Although soybean is a major source of essential amino acids in poultry diets, the presence of antinutritional factors, some of them thermo-resistant and others thermolabile, limits the use of soybean in poultry diets and hinders its inclusion as a raw ingredient (10). SBA is one of the major antinutritional factors present in soybeans (10). As SBA is a thermolabile molecule, it can be reduced but not completely inactivated during the roasting process of soybean (11, 12). SBA is also resistant to the action of proteolytic enzymes (12, 13).

SBA has a highly specific binding affinity for *N*-Acetyl-D-galactosamine, a carbohydrate present in the cell membrane of enterocytes (14). It binds to the small intestinal epithelium, mainly at the brush border, causing morphological changes in these cells, such as microvillar atrophy and hyperplasia (9). Upon binding, SBA acts as a chemical messenger, initiating an inflammatory response and inducing cytotoxic damage to the enterocytes (15). The above is relevant to the feeding of young stock (chicks or jakes) in the poultry industry or piglets since the presence of SBA alters the digestive process, the feeding efficiency, growth, and health status of the animals by impairing cell proliferation and permeability and cell signaling pathways, ultimately inducing apoptosis and autophagy (16). All these lesions affect the barrier function of the small intestinal mucosa, the absorption and transport of nutrients across the intestinal wall, and the reduction of enzymes such as hydrolases; they also destroy endocrine cells and, consequently, the reduction of gut hormones (14, 15).

IgY technology, based on the immunization of chickens and the extraction and purification of specific antibodies from their egg yolks, has been used in the development of products for the prevention or treatment of various pathologies or health conditions in animals and humans (17). This technology has been increasingly studied and applied in the last two decades in several contexts of animal production and animal health, proving to be low-cost, animal welfare-friendly, and more efficient compared to the traditional production of antibodies from live animals (18).

Against this background, this study aimed to produce anti-SBA IgY and evaluate its potential to neutralize the biological activity of SBA *in vitro*.

## Materials and methods

### Ethics statement

All procedures were reviewed and approved by the Bioethics Committee (Certification N° 10-21) of the Catholic University of Temuco, Chile and were performed in accordance with the animal care protocols established by the institution.

### Production of egg yolk antibodies

#### Immunization of hens

Lohmann Brown laying hens (*n* = 10; 35 weeks old) were individually housed in wire cages with a light/dark cycle of 17/7 h and a room temperature ranging from 15 to 20°C for 3 months. Hens were given *ad libitum* access to water and a commercial feed and were acclimated to the environment and handling for 2 weeks prior to the start of the experiment. Hens in the treatment group (*n* = 5) were immunized 4 times every 15 days (days 0, 15, 30, and 45). For the first immunization, 100 μg of soybean lectin (SBA; Sigma- Aldrich, USA) was emulsified with Freund's complete adjuvant and injected intramuscularly into the pectoral muscles at 4–5 different injection sites (0.5 ml/hen), as suggested by several authors (17, 19, 20). Booster doses consisting of 50 μg SBA emulsified with Freund's incomplete adjuvant were administered by the same route (20). Hens in the control group (*n* = 5) were injected with 0.5 ml of saline per hen, using the same procedure described for the treatment group. Before the first immunization and 2 weeks after each immunization (days 0, 15, 30, 45, and 60), blood samples were collected from the cubital vein of all hens and transferred to 2 ml Eppendorf tubes. Blood was held at room temperature for 12 h, and serum samples were collected by centrifugation after clotting and frozen at −20°C until analysis. After the last immunization, eggs were collected from both groups for 15 days and stored separately at room temperature (15–18°C) until processing.

#### IgY isolation and purification

IgY was extracted from a pool of randomly selected eggs, separately for the treatment and control groups (*n* = 10 eggs/group), using the polyethylene glycol (PEG) 6000 precipitation method described by Pauly et al. (18). In our experience, once standardized, this method of IgY extraction is very efficient, and the IgY samples obtained performed well in various immunological assays (18). For each group, the egg yolk pool was diluted at a ratio of 1:2 in PBS (pH 7.2), precipitated with 3.5% (w/v) PEG, and centrifuged at 4°C for 20 min at 10,000 rpm. The delipidated supernatant was filtered and subjected to two further precipitations with 8.5 and 12% PEG. The final pellet was diluted in 800 μl PBS and transferred into a tubular dialysis membrane (PM 14 kDa, Sigma-Aldrich, USA). The extract was dialysed against 0.017 M NaCl for 12 h at room temperature, diluted in PBS, and subjected to a second dialysis under the same conditions. The total protein content of the final extract was measured by the bicinchoninic acid (BCA) method using a commercial kit (Pierce^TM^ BCA Protein Assay, USA).

#### Determination of the extract purity and specificity of anti-SBA IgY

The protein profile of the egg yolk extracts from the treatment and control groups was analyzed by SDS-PAGE under reducing conditions and stained with Coomassie blue 25% (blue R250, Sigma-Aldrich, USA). A molecular weight marker (range 11–245 kDa; Page Ruler Plus Prestained Protein Ladder, Thermofisher Scientific) and a commercially purified IgY standard (Sigma-Aldrich, USA) were included. Proteins were then electro-transferred to polyvinylidene difluoride membranes (PVDF, Thermo Fisher Scientific, USA) using a Mini Trans-Blot cell system (Bio-Rad, USA). Membranes were incubated with anti-IgY (heavy chain) conjugated to peroxidase (Jackson ImmunoResearch 303-0035-008, USA) and detected with diaminobenzidine (DAB, Thermofisher Scientific, USA).

Specific detection of the SBA by the obtained IgY was also performed using Western blotting. The SBA (Sigma-Aldrich, USA) was subjected to an SDS-PAGE run and electroblotted onto PVDF membranes. After blocking with 3% milk, the membrane was incubated at 37°C for 1 h with the IgY extracts from the treatment and control groups at 1:1.000 dilution in PBS. After three washes, the membrane was incubated at 37°C for 1 h with an anti-chicken IgY-peroxidase antibody at 1:5.000 dilution in PBS. Finally, the membranes were developed using DAB (Thermofisher Scientific, USA).

#### The quantification of IgY in blood serum and egg yolk

The quantification of IgY in serum and egg yolk from immunized and control hens was performed by indirect ELISA. Microplates (Maxisor; Thermo Scientific, USA) were coated with 1 μg/well of purified SBA in 100 μl of PBS and incubated at 20°C for 12 h. The microplates were then washed three times with PBS, then blocked with 2% skimmed milk at 37°C for 1 h, and washed again. Serial dilutions of the sera and the yolk extracts were made with PBS-Tween, and 100 μl of the diluted extracts were added in duplicate to the wells and incubated at 37°C for 1 h. The wells were then washed with PBS-Tween, and 100 μl of anti-IgY antibody conjugated to peroxidase (Jackson ImmunoResearch 303-0035-008, USA; dilution 1:140.000) was added to each well. After 1 h of incubation, the wells were washed again with PBS-Tween, and 100 μl of the tetramethylbenzidine substrate solution (TMB, Thermo Fisher, USA) was added. After 15 min, 50 μl of 1 M phosphoric acid (Merck, USA) was added to stop the reaction. Absorbance was measured at 450 nm using a microplate reader (Sunostik-SPR-960, Sunostik Medical Technology, China).

### *In vitro* inhibition of SBA haemagglutinating activity by anti-lectin IgY

#### Haemagglutination

SBA-induced haemagglutination reaction was performed according to Spackman (21). Blood samples (type A) were obtained from volunteers within our research group after signing an informed consent form. Blood was collected in 5 ml tubes containing EDTA and centrifuged at 4.000 rpm for 10 min to obtain plasma. The erythrocytes were washed four times in saline and then resuspended in the same solution (2% v/v) until use. Haemagglutination was performed in *U*-shaped multiwell plates (Nunc^TM^, Thermo Fisher, USA). In total, 50 μl of PBS was added to all wells. The first well in each row was used as the control. A total of 50 μl of SBA (Sigma-Aldrich, USA; 1 μg/μ) was added to the second well of each row. Serial dilutions were then performed, and 50 μl of erythrocyte solution (2%) was added to all the wells. The microplate was then gently vortexed and incubated at 20°C for 2 h. All the samples were tested in duplicate. The haemagglutination response observed corresponded to 512 units of haemagglutination/50 μl (UHA/50 μl). One UHA/50 μl was defined as the reciprocal of the maximum dilution, giving positive haemagglutination (21).

#### Inhibition of haemagglutination

For the inhibition of haemagglutination, 50 μl of PBS and 98 ng of SBA (the amount of SBA present in the maximal positive haemagglutination dilution; 512 UHA/50 μl) were added to all the wells of the microplate. The inhibitory activity of serially diluted D-galactose (Sigma-Aldrich, USA; 350 μg/50μL), IgY from non-immunized chickens (1.250 μg/50 μL), and anti-SBA IgY (1.250 μg/50 μL) from immunized chickens were measured after the addition of 50 μl of erythrocyte solution (2%) to each well. The microplates were incubated at 20°C for 2 h. All samples were assayed in duplicate. Inhibition of haemaggutination results were expressed as units of haemagglutination inhibition (UHI), which is defined as the reciprocal of the maximum dilution of the tested solutions that completely inhibited haemagglutination (21).

### Statistical analysis

Blood serum IgY titres in immunized and non-immunized hens were compared using a Statistical Analysis System (SAS, version 9.1.2, 2004; SAS Institute, Cary, NC, USA) with the mixed procedure for repeated measures. The model included treatment, time, and treatment-by-time interaction using time as the repeated variable. IgY concentrations in blood serum and egg yolk were compared between groups using Student's *t*-test. Values are expressed as mean ± SD. The *p*-values of ≤0.05 were considered significant.

## Results

**Figure 1:**
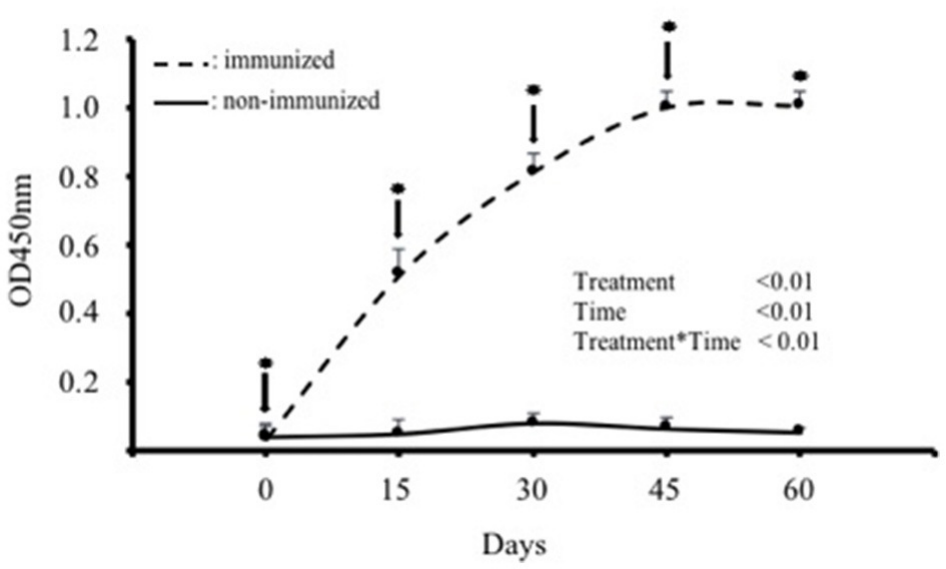
IgY antibody levels in the blood serum of hens immunized with purified soybean lectin (SBA; treatment group; *n* = 5) or saline (control group; *n* = 5). Absolute absorbance values (OD at 450 nm) are expressed as mean ± SD. ↓ indicates the day of immunization or saline administration, and * indicates the day of blood collection for both groups. ODs on day 0 correspond to the first sample taken before the first immunization.

IgY titres in the blood serum of the hens immunized with SBA increased significantly (*p* < 0.01) after the first immunization and subsequent boosters, reaching a plateau between days 45 and 60. The mean maximum IgY titer was observed 45 days after the first immunization (Figure 1). In contrast, IgY titres in the control group remained at basal levels throughout the evaluation period (Figure 1).

The protein content of the egg yolk extract from immunized and non-immunized hens was 38.7 ± 1.6 and 37.7 ± 1.5 mg/ml, respectively. Since the average volume of the egg yolk extract obtained from each egg was 2.5 ml, the total amount of protein obtained from each egg was 96.8 ± 4.0 and 94.8± 3.0 mg for immunized and non-immunized hens, respectively.

The specific anti-SBA Ig Y titer detected by ELISA in the yolk extracts was significantly higher (*p* < 0.001) in immunized hens than in the non-immunized control group, with OD_450nm_ values of 0.98 ± 0.05 and 0.058 ± 0.02, respectively.

**Figure 2:**
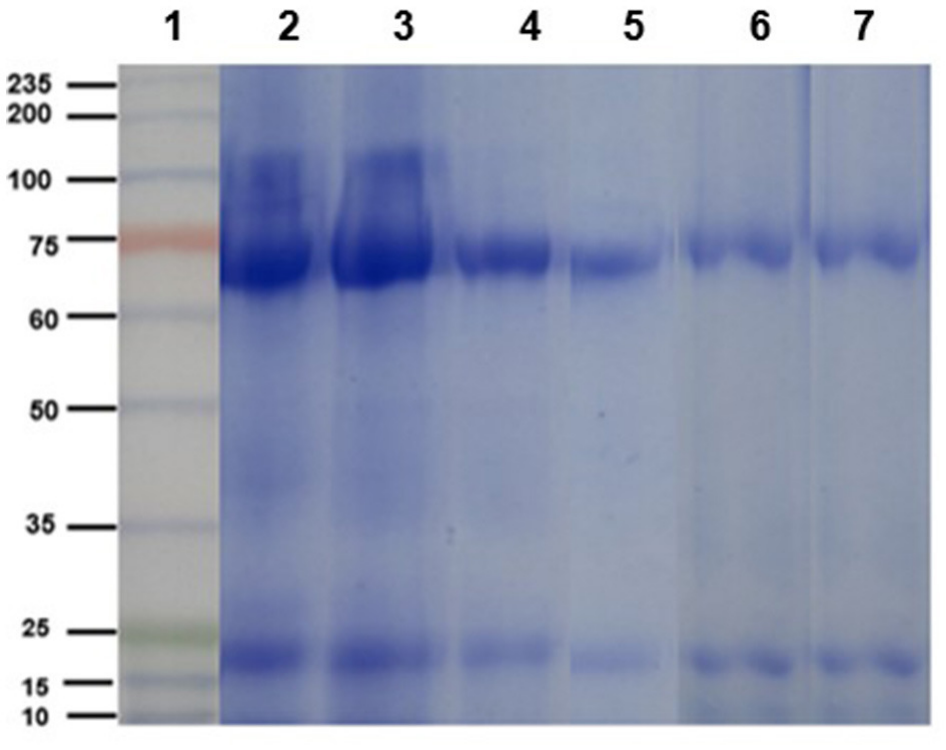
Electrophoretic profile (SDS-PAGE 10% under reducing conditions) of chicken IgY. Lane 1: molecular weight ladder. Lanes 2 and 3: purified IgY (control sample) heavy and light chains. Lanes 4 and 5: egg yolk protein extracts from immunized hens (*n* = 5). Lanes 6 and 7: egg yolk protein extracts from non-immunized hens (*n* = 5).

The electrophoretic pattern of the egg yolk extract from immunized and non-immunized hens consisted of two bands of ~65 y ~27 kDa, corresponding to the molecular weight of the heavy and light chains of IgY, as can be contrasted with the commercial IgY (control, Figure 2).

**Figure 3:**
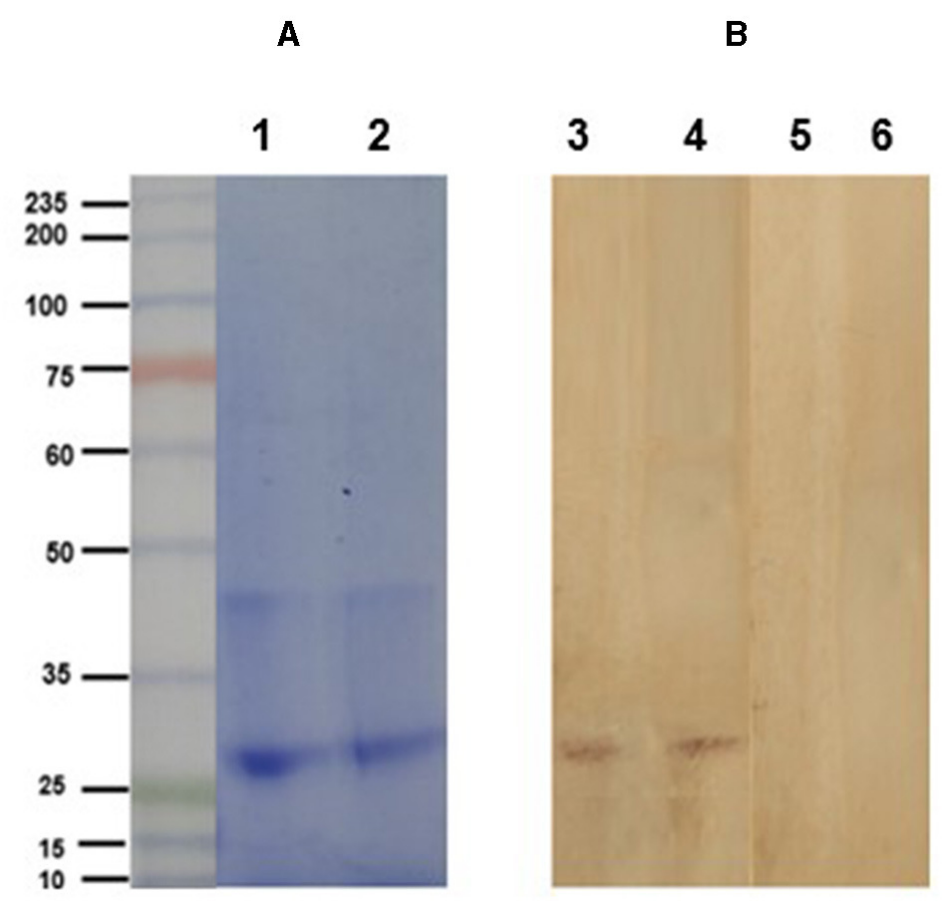
**(A)** Electrophoretic profile (SDS-PAGE 10% under reducing conditions) of purified soybean lectin (SBA; Sigma-Aldrich, USA; lanes 2 and 3). Lane 1: molecular weight ladder. **(B)** Immunoblot analysis of soybean lectin incubated with IgY from the treatment (lanes 1 and 2) and control (lanes 3 and 4) groups, using a rabbit anti-chicken IgY peroxidase antibody.

The electrophoretic pattern of the soybean lectin consisted of a band at 30 kDa (Figure 3). Western blot clearly shows the specific detection of the soybean agglutinin (30 kDa band) by the anti-SBA IgY. In contrast, agglutinin was not detected by the IgY obtained from eggs of non-immunized hens (Figure 3).

The inhibition of SBA-induced haemagglutination by the anti-SBA IgY reached 512 UHI, compared with 8 UHI and 256 UHI, respectively, when IgY was obtained from eggs of control hens or when D-galactose (positive control) was used (Table 1). The magnitude of inhibition of haemagglutination induced by D-galactose and non-specific IgY from the control group was 50 and 1.6%, respectively, of that obtained when anti-SBA IgY antibodies were used (Table 1).

**Table 1:** The inhibition of SBA-induced haemagglutination by D-galactose (positive control) or IgY obtained from egg yolks of immunized (treatment group; *n* = 5) and non-immunized hens (control group; *n* = 5).

	**Inhibition of haemagglutination**	
	**(UHI** ^*^ **)**	**(%)**
Anti-SBA IgY (treatment group)	512	100
Non-specific IgY (control group)	8	1.6
D-galactose	256	50

The inhibition of haemagglutination is expressed in the form of units (UHI) and percentage (%).

^*^UHI is defined as the reciprocal of the maximum dilution of the tested solutions that completely inhibits haemagglutination.

## Discussion

Our results show that it is possible to produce high concentrations of anti-SBA antibodies using IgY technology, which effectively inhibits the haemagglutination capacity of SBA in *in vitro* assays. This antinutritional factor of the soybean has been overlooked and only superficially studied with respect to its deleterious effects on poultry and pig feeding efficiency (22). SBA specifically binds with high affinity to cell receptors *N*-Acetyl-D-galactosamine present in the enterocyte membrane of poultry and mammals, causing deleterious morphological changes due to an induced inflammatory response (22). All of the morphological changes induced by SBA in the small intestinal mucosa result in enzymatic inhibition at the brush border, which alters digestion and absorption of nutrients and ultimately adversely affects the growth and health status of the animals (9, 23). In addition, severe negative effects on the local immune system have been reported (16, 24).

Our immunization protocol (i.e., four immunisations every 15 days) induced a rapid and sustained immune response in the hens, resulting in an abrupt and significant increase in blood anti-SBA IgY after the first immunization. In this regard, it has been described that some hens respond with mammalian-like antibody kinetics, while other birds may respond with a rapid rise in antibody titer after the first immunization (25), as observed in our assays. Hens in the immunized group maintained high IgY titres until the end of the evaluation period, reflecting a beneficial effect of booster immunisations on increasing IgY antibody titres, as previously described (26). Similar immune responses have been reported in hens following immunization with various bacterial antigens, such as *Escherichia coli* (27), *Campylobacter jejuni* (28), *Salmonella typhimurium* (29), fungi (30), viruses (31), parasites (32), or viper (33), and honeybee venom (34).

The total IgY concentration (i.e., total protein concentration) in the egg yolk extract was higher than previously reported by other authors (32, 34–36) who described protein concentrations ranging from 5.4 to 33 mg/ml. The differences in total protein extracted in these reports compared to the present study may be due to the extraction technique used (37). On the other hand, the similar total IgY concentration in egg yolk from immunized and non-immunized hens is explained by the fact that only 1–10% of the total IgY present is specific (20).

The recovery rate of total protein per egg was also higher than previously reported by Barroso et al. (37) and Carroll and Stollar (38). Similarly, Shin et al. (39) and Sunwoo et al. (40) highlight that the IgY concentration in the egg yolk ranges from 9.4 to 133 mg/ml, depending on the extraction technique used (37). This finding suggests that the use of PEG may maximize the extraction of IgY, although scaling up of this method may be more complex than other methods described for the purification of the immunoglobulin from the egg yolk, as discussed by Pereira et al. (17).

On the other hand, collecting the eggs 10 days after the last immunization could provide the highest concentration of specific IgY in the yolk due to the strong correlation between the immunoglobulin in the yolk and the blood (41, 42).

The specificity of the anti-SBA IgY produced in this study was demonstrated by Western blotting and the haemagglutination inhibition (HI) test, where anti-SBA IgY blocked SBA-induced haemagglutination up to a high dilution. This finding suggests that the specific IgY could efficiently bind to the active sites of SBA, probably by blocking its association with the surface of the erythrocytes. As IgY are polyclonal antibodies, they can recognize and bind to different epitopes of a single antigen (43). These antibodies may also lead to conformational changes, in addition to the epitope binding mechanism, that could enhance the inactivation of the SBA haemagglutination activity. This finding is supported by the high percentage of inhibition of SBA haemagglutination activity observed with the specific IgY compared not only to IgY from non-immunized hens but also to D-galactose, which was considered as a positive inhibition control in the assays.

To date, the detrimental antinutritional effects of SBA in animal diets have been underestimated. It has been found that low levels of SBA, even as low as 0.5–2 mg/g of soybean meal for chicks and piglets, can cause alterations in enterocyte morphology and growth rate (14). However, few strategies have been proposed to reduce the level of SBA in the final animal diet. Some of these strategies include (a) the development of SBA-free soybean varieties (44), (b) the addition of oligosaccharides (12) or *N*-acetylgalactosamine (45) to the animal diet, or (c) the supplementation of diets with probiotic bacteria expressing *N*-acetylgalactosamine in their surface (46). However, none of these strategies seem fully effective and thus have not been implemented in the industry.

IgY technology has been extensively studied and applied to various aspects of animal and human health (17). The use of specific IgY antibodies has been repeatedly reported in different animal species as a highly effective prophylactic strategy to confer passive immunity to animals, neutralize bacterial toxins, and inhibit bacterial adhesion to cells or block enzymes from various bacteria {[*Escherichia coli*; (47)], [*Campylobacter jejuni*; (48)], and [*Salmonella Typhimurium and Salmonella Enteritidis*, (49)]}. Specific IgY has also been used against other infectious agents such as fungi (30), parasites (50), and viruses (51), or molecules present in venom/toxins from snakes, scorpions, and bees (34, 52, 53). All these previous studies strongly support and encourage the use of IgY technology against antinutritional factors present in animal feed, especially those that have shown positive results after oral IgY administration.

Based on our results, we can conclude that anti-SBA IgY antibodies can be efficiently produced at high concentrations and are specific and effective in inhibiting SBA-induced haemagglutination *in vitro*, doubling the inhibitory capacity of D-galactose. In this context, administering anti-SBA IgY in the diets, for example, as an egg yolk powder additive, may be an effective alternative to reduce the impact of the antinutritional adverse effects of diets formulated with soybean meal. Future trials should evaluate the addition of anti-SBA IgY to the diets of chicks and piglets, as these categories of animals are more susceptible to the detrimental effects of SBA on their intestinal morphology and function.

## Data availability statement

The raw data supporting the conclusions of this article will be made available by the authors, without undue reservation.

## Ethics statement

The studies involving humans were approved by Comité de Bioética, Universidad Católica de Temuco. The studies were conducted in accordance with the local legislation and institutional requirements. The human samples used in this study were acquired from Voluntary donation of blood sample by members of the research group. Written informed consent for participation was not required from the participants or the participants' legal guardians/next of kin in accordance with the national legislation and institutional requirements. The animal study was approved by Comité de Bioética, Universidad Católica de Temuco. The study was conducted in accordance with the local legislation and institutional requirements.

## Author contributions

NR: Conceptualization, Data curation, Formal analysis, Funding acquisition, Investigation, Methodology, Project administration, Resources, Software, Supervision, Validation, Visualization, Writing—original draft, Writing—review & editing. CC: Conceptualization, Formal analysis, Investigation, Methodology, Writing—review & editing. MS: Investigation, Methodology, Supervision, Writing—original draft. AH: Data curation, Formal analysis, Investigation, Methodology, Writing—review & editing. PC: Investigation, Writing—original draft, Data curation, Formal analysis, Methodology.

## References

[1] Cavada BS Osterne VJ Oliveira MV Pinto-Junior VR Silva MT Bari AU. Reviewing Mimosoideae lectins: a group of under explored legume lectins. Int J Biol Macromol. (2020) 154:159–65. 10.1016/j.ijbiomac.2020.03.113 32184140

[2] Grandhi NJ Mamidi AS Surolia A. Pattern recognition in legume lectins to extrapolate amino acid variability to sugar specificity. Adv Exp Med Biol. (2015) 842:199–215. 10.1007/978-3-319-11280-0_13 25408345

[3] Chatterjee M Mandal DK. Kinetic analysis of subunit oligomerization of the legume lectin soybean agglutinin. Biochemistry. (2003) 42:12217–22. 10.1021/bi034642l 14567683

[4] Laija SN Mahesh S Smitha LS Remani P. Isolation and partial characterization of two plant lectins. Curr Res J Biol Sci. (2010) 2:232−7.

[5] Lotan R Siegelman HW Lis H Sharon N. Subunit structure of soybean agglutinin. J Biol Chem. (1974) 249:1219–24. 10.1016/S0021-9258(19)42963-3 4855992

[6] Sinha S Mitra N Kumar G Bajaj K Surolia A. Unfolding studies on soybean agglutinin and concanavalin A tetramers: a comparative account. Biophys J. (2005) 88:1300–10. 10.1529/biophysj.104.051052 15542553 PMC1305132

[7] Halder S Surolia A Mukhopadhyay C. Dynamics simulation of soybean agglutinin (SBA) dimer reveals the impact of glycosylation on its enhanced structural stability. Carbohydr Res. (2016) 428:8–17. 10.1016/j.carres.2016.04.009 27108103

[8] Vodkin LO Raikhel NV. Soybean lectin and related proteins in seeds and roots of le and le soybean varieties. Plant Physiol. (1986) 81:558–65. 10.1104/pp.81.2.558 16664856 PMC1075376

[9] Fasina YO Classen HL Garlich JD Black BL Ferket PR Uni Z. Response of turkey poults to soybean lectin levels typically encountered in commercial diets. 2 Effect on intestinal development and lymphoid organs. Poult Sci. (2006) 85:870–7. 10.1093/ps/85.5.870 16673765

[10] Liu K and Ruiz N. Soybean trypsin inhibitor and urease activities and their correlations as affected by heating method, duration, sample matrix, and prior soaking. J Am Oil Chem Soc. (2021) 98:795–810. 10.1002/aocs.12514

[11] Fasina YO Classen HL Garlich JD Swaisgood HE Clare DA. Investigating the possibility of monitoring lectin levels in commercial soybean meals intended for poultry feeding using steam-heated soybean meal as a model. Poult Sci. (2003) 82:648–56. 10.1093/ps/82.4.648 12710487

[12] Pan L Farouk MH Qin G Zhao Y Bao N. The influences of soybean agglutinin and functional oligosaccharides on the intestinal tract of monogastric animals. Int J Mol Sci. (2018) 19:554. 10.3390/ijms19020554 29439523 PMC5855776

[13] Muramoto K. Lectins as bioactive proteins in foods and feeds. Food Sci Technol Res. (2017) 23:487–94. 10.3136/fstr.23.487

[14] Pan L Qin G Zhao Y Wang J Liu F Che D. Effects of soybean agglutinin on mechanical barrier function and tight junction protein expression in intestinal epithelial cells from piglets. Int J Mol Sci. (2013) 14:21689–704. 10.3390/ijms141121689 24189218 PMC3856029

[15] Babot JD Argañaraz-Martínez E Lorenzo-Pisarello MJ Apella MC Perez Chaia A. Cytotoxic damage of soybean agglutinin on intestinal epithelial cells of broiler chicks: *in vitro* protection by Bifidobacterium infantis CRL1395. FEMS Microbiol Lett. (2016) 363:114. 10.1093/femsle/fnw114 27190155

[16] Lagarda-Diaz I Guzman-Partida A Vazquez-Moreno L. Legume lectins: proteins with diverse applications. Int J Mol Sci. (2017) 18:1242. 10.3390/ijms18061242 28604616 PMC5486065

[17] Pereira EP van Tilburg MF Florean EO Guedes MI. Egg yolk antibodies (IgY) and their applications in human and veterinary health: a review. Int Immunopharmacol. (2019) 73:293–303. 10.1016/j.intimp.2019.05.015 31128529 PMC7106195

[18] Pauly D Chacana PA Calzado EG Brembs B Schade R. IgY technology: extraction of chicken antibodies from egg yolk by polyethylene glycol (PEG) precipitation. J Vis Exp. (2011) 51:3084. 10.3791/3084-v 21559009 PMC3197133

[19] Cook IF. Subcutaneous vaccine administration - an outmoded practice. Hum Vaccin Immunother. (2021) 17:1329–41. 10.1080/21645515.2020.1814094 32991241 PMC8086591

[20] Schade R Calzado EG Sarmiento R Chacana PA Porankiewicz-Asplund J Terzolo HR. Chicken egg yolk antibodies (IgY-technology): a review of progress in production and use in research and human and veterinary medicine. Altern Lab Anim. (2005) 33:129–54. 10.1177/026119290503300208 16180988

[21] Spackman E Sitaras I. Hemagglutination inhibition assay. Methods Mol Biol. (2020) 2123:11–28. 10.1007/978-1-0716-0346-8_2 32170677

[22] Pan L Liu Y Lan H Bao N Zhao Y Sun H. Biological mechanisms induced by soybean agglutinin using an intestinal cell model of monogastric animals. Front Vet Sci. (2021) 8:639792. 10.3389/fvets.2021.639792 34150879 PMC8207199

[23] Salgado P Freire JP Mourato M Cabral F Toullec R Lallès JP. Comparative effects of different legume protein sources in weaned piglets: Nutrient digestibility, intestinal morphology, and digestive enzymes. Livest Prod Sci. (2002) 74:191–202. 10.1016/S0301-6226(01)00297-4

[24] Röhe I Göbel TW Goodarzi Boroojeni F Zentek J. Effect of feeding soybean meal and differently processed peas on the gut mucosal immune system of broilers. Poult Sci. (2017) 96:2064–73. 10.3382/ps/pew491 28339726

[25] Patterson R Youngner JS Weigle WO Dixon FJ. Antibody production and transfer to egg yolk in chickens. *J Immunol*. (1962) 89:272–8. 10.4049/jimmunol.89.2.272 14484407

[26] Correia-Neto C Oliveira M Silva M Pinto-Junior V Correia S Silva I. Anti-vatairea guianensis lectin Igy antibodies produced in immunized chicken eggs: production, isolation and characterization. Int J Dev Res. (2020) 10:41810–14.

[27] Fathi J Ebrahimi F Nazarian S Hajizade A Malekzadegan Y Abdi A. Production of egg yolk antibody (IgY) against shiga-like toxin (stx) and evaluation of its prophylaxis potency in mice. Microb Pathog. (2020) 145:104199. 10.1016/j.micpath.2020.104199 32320733

[28] Hermans D Van Steendam K Verbrugghe E Verlinden M Martel A Seliwiorstow T. Passive immunization to reduce *Campylobacter jejuni* colonization and transmission in broiler chickens. Vet Res. (2014) 45:27. 10.1186/1297-9716-45-27 24589217 PMC3996517

[29] Sunwoo HH Nakano T Dixon WT Sim JS. Immune responses in chickens against lipopolysaccharide of *Escherichia coli* and *Salmonella typhimurium*. *Poult Sci*. (1996) 75:342–5. 10.3382/ps.0750342 8778727

[30] de Souza PC Corrêa AE Gameiro JG de Oliveira Júnior AG Panagio LA Venancio EJ. Production of IgY against iron permease Ftr1 from *Candida albicans* and evaluation of its antifungal activity using *Galleria mellonella* as a model of systemic infection. Microb Pathog. (2023) 181:106166. 10.1016/j.micpath.2023.106166 37290729

[31] Chen CJ Hudson AF Jia AS Kunchur CR Song AJ Tran E. Tran E, Affordable IgY-based antiviral prophylaxis for resource-limited settings to address epidemic and pandemic risks. J Glob Health. (2022) 12:05009. 10.7189/jogh.12.05009 35265332 PMC8877785

[32] Grando TH Baldissera MD de Sá MF do Carmo GM Porto BC Aguirre GS. Avian antibodies (IgY) against Trypanosoma cruzi: Purification and characterization studies. J Immunol Methods. (2017) 449:56–61. 10.1016/j.jim.2017.07.002 28697990 PMC7126890

[33] Lee CH Liu CI Leu SJ Lee YC Chiang JR Chiang LC. Chicken antibodies against venom proteins of *Trimeresurus stejnegeri* in Taiwan. J Venom Anim Toxins Incl Trop Dis. (2020) 26:e20200056. 10.1590/1678-9199-jvatitd-2020-0056 33281887 PMC7682652

[34] Leiva CL Geoghegan P Lammer M Cangelosi A Mariconda V Celi AB. *In vivo* neutralization of bee venom lethality by IgY antibodies. Mol Immunol. (2021) 135:183–90. 10.1016/j.molimm.2021.04.015 33930713

[35] Sunwoo HH Wang WW Sim JS. Detection of *Escherichia coli* O157:H7 using chicken immunoglobulin Y. Immunol Lett. (2006) 106:191–3. 10.1016/j.imlet.2006.05.005 16781781

[36] Ren H Yang W Thirumalai D Zhang X Schade R. A comparative evaluation of six principal IgY antibody extraction methods. Altern Lab Anim. (2016) 44:11–20. 10.1177/026119291604400111 27031600

[37] Barroso P Murcia H Vega N Pérez G. Purification of IgY against Salvia bogotensis lectin. Biomedica. (2005) 25:496–510. 10.7705/biomedica.v25i4.1376 16433176

[38] Carroll SB Stollar BD. Antibodies to calf thymus RNA polymerase II from egg yolks of immunized hens. J Biol Chem. (1983) 258:24–6. 10.1016/S0021-9258(18)33211-3 6336747

[39] Shin JH Yang M Nam SW Kim JT Myung NH Bang WG. Use of egg yolk-derived immunoglobulin as an alternative to antibiotic treatment for control of *Helicobacter pylori* infection. Clin Diagn Lab Immunol. (2002) 9:1061–6. 10.1128/CDLI.9.5.1061-1066.2002 12204960 PMC120060

[40] Sunwoo HH Lee EN Menninen K Suresh MR Sim JS. Growth inhibitory effect of chicken egg yolk antibody (IgY) on *Escherichia coli* O157:H7. J Food Sci. (2002) 67:1486–94. 10.1111/j.1365-2621.2002.tb10310.x

[41] Loeken MR Roth TF. Analysis of maternal IgG subpopulations which are transported into the chicken oocyte. Immunology. (1983) 49:21–8. 6840806 PMC1454086

[42] Hamal KR Burgess SC Pevzner IY Erf GF. Maternal antibody transfer from dams to their egg yolks, egg whites, and chicks in meat lines of chickens. Poult Sci. (2006) 85:1364–72. 10.1093/ps/85.8.1364 16903465

[43] Seixas AM Sousa SA Leitão JH. Antibody-based immunotherapies as a tool for tackling multidrug-resistant bacterial infections. Vaccines. (2022) 10:1789. 10.3390/vaccines10111789 36366297 PMC9695245

[44] Douglas MW Parsons CM Hymowitz T. Nutritional evaluation of lectin-free soybeans for poultry. Poult Sci. (1999) 78:91–5. 10.1093/ps/78.1.91 10023754

[45] Zhao B Che D Adams S Guo N Han R Zhang C. N-acetyl-D-galactosamine prevents soya bean agglutinin-induced intestinal barrier dysfunction in intestinal porcine epithelial cells. J Anim Physiol Anim Nutr. (2019) 103:1198–206. 10.1111/jpn.13091 30934149

[46] Babot JD Argañaraz-Martínez E Quiroga M Grande SM Apella MC Perez Chaia A. Protection of the intestinal epithelium of poultry against deleterious effects of dietary lectins by a multi-strain bacterial supplement. Res Vet Sci. (2021) 135:27–35. 10.1016/j.rvsc.2020.12.020 33422729

[47] Li XY Jin LJ Uzonna JE Li SY Liu JJ Li HQ. Chitosan-alginate microcapsules for oral delivery of egg yolk immunoglobulin (IgY): *in vivo* evaluation in a pig model of enteric colibacillosis. Vet Immunol Immunopathol. (2009) 129:132–6. 10.1016/j.vetimm.2008.12.016 19150135

[48] Thibodeau A Fravalo P Perron A Lewandowski SL Letellier A. Production and characterization of anti-*Campylobacter jejuni* IgY derived from egg yolks. Acta Vet Scand. (2017) 59:80. 10.1186/s13028-017-0346-4 29208016 PMC5717825

[49] Chalghoumi R Théwis A Beckers Y Marcq C Portetelle D Schneider YJ. Adhesion and growth inhibitory effect of chicken egg yolk antibody (IgY) on *Salmonella enterica* serovars Enteritidis and Typhimurium *in vitro*. Foodborne Pathog Dis. (2009) 5:593–604. 10.1089/fpd.2008.0258 19388827

[50] Xu J Ren C Wang S Liu D Cao L Tao J. Protection efficacy of multivalent egg yolk immunoglobulin against *Eimeria tenella* infection in chickens. Iran J Parasitol. (2013) 8:449–58. 24454440 PMC3887248

[51] Vega CG Bok M Vlasova AN Chattha KS Fernández FM Wigdorovitz A. IgY antibodies protect against human Rotavirus induced diarrhea in the neonatal gnotobiotic piglet disease model. PLoS ONE. (2012) 7:e42788. 10.1371/journal.pone.0042788 22880110 PMC3411843

[52] da Rocha DG Fernandez JH de Almeida CM da Silva CL Magnoli FC da Silva OÉ. Development of IgY antibodies against anti-snake toxins endowed with highly lethal neutralizing activity. Eur J Pharm Sci. (2017) 106:404–12. 10.1016/j.ejps.2017.05.069 28595875

[53] Sifi A Adi-Bessalem S Laraba-Djebari F. Development of a new approach of immunotherapy against scorpion envenoming: avian IgYs an alternative to equine IgGs. Int Immunopharmacol. (2018) 61:256–65. 10.1016/j.intimp.2018.06.013 29902709

